# Characterization of Changes in Subchondral Bone Tissue Density of the Ankle Joint in Taekwondo Players

**DOI:** 10.3389/fbioe.2022.872258

**Published:** 2022-05-04

**Authors:** Guanghua Xu, Hongyu Liu, Lifu Zhang

**Affiliations:** Institute of Sport and Exercise Medicine, North University of China, Taiyuan, China

**Keywords:** Taekwondo, distal tibial, talus dome, bone density distribution, bone remodeling, computed tomography osteoabsorptiometry

## Abstract

**Background:** It has been found that ankle joint impingement can cause articular cartilage injury, and the change of subchondral bone density and distribution under long-term stress loading can reflect the stress interaction of the articular surface and the difference in bone remodeling degree and predict the location of cartilage injury.

**Objective:** To investigate the bone density distribution pattern of ankle joint subchondral bone under mechanical stress loading of Taekwondo, the volume proportion of bone tissue with different bone densities, and the distribution characteristics of bone remodeling position.

**Study design:** A controlled laboratory study.

**Methods:** Computed tomography data were collected from the feet of 10 normal subjects (control group) and 10 high-level Taekwondo athletes. First, the distribution pattern of the high-density area of the articular surface was determined by computed tomography osteoabsorptiometry and the nine-grid anatomical region localization method. Second, the percentage of bone volume (%BTV) and the distribution trend of bone tissue were measured.

**Result:** In the present study, it was found that there were high-density areas in the 1st, 2nd, 3rd, 4th, 6th, 7th, and 9th regions of the distal tibia of Taekwondo athletes, and the distribution track was consistent with the high-density areas of the talar dome surface (1st, 2nd, 3rd, 4th, 6th, 7th, and 9th regions). In Taekwondo athletes, the percentage of bone tissue volume in the distal tibia and talus with high and moderate bone density was significantly higher than that in the control group (*p* < 0.05).

**Conclusion:** The impact stress, ground reaction force, intra-articular stress, lower limb movement technology, lower limb muscle, and tendon stress caused by Taekwondo lead to special pressure distribution patterns and bone tissue remodeling in the ankle.

## Introduction

Taekwondo sports technology is complex and diverse. In particular, Taekwondo athletes need to use their bodies to obtain effective hitting scores by rotating their trunks and hitting their opponent’s head and chest (Watta, 2021) with their feet. This means that the lower limbs of Taekwondo athletes may be affected by mechanical factors: impact stress and ground reaction force after jumping and landing. In addition, the technical characteristics of Taekwondo lower limbs are strong leg strength, fast-hitting speed, flexible footwork, and hitting opponents in movement. The technical movements of Taekwondo include lower limb hitting techniques such as roundabout kick, side kick, back kick, down split kick, and push kick. According to the statistics of researchers, 98% of the scores of competitive Taekwondo athletes in Taekwondo competitions come from leg blows (Jakubiak and Saunders, 2008). This shows that the score of Taekwondo depends on the hitting of lower limbs; the more difficult the hitting action is, the more concentrated it is on the head, and the higher the hitting score will be obtained.

Early studies conducted kinematic analysis on the lower limb kicking of Taekwondo athletes and found that the impact force of Taekwondo athletes’ roundhouse kick is about 1,000 N–3,000 N. The impact force of contestants is higher than that of noncontestants, and the leg impact stress of nonmedal contestants is far lower than that of medal winners (Falco et al., 2009; Estevan et al., 2011; Thibordee and Prasartwuth, 2014). However, there were also studies that Taekwondo athletes need a certain vertical take-off height before hitting (Pieter et al., 2012), but Taekwondo athletes must accept the ground reaction force of 6 times their body weight after taking off and hitting the target and landing. If one leg lands barefoot, it may further increase the risk of lower limb injury, and the reaction force changes with the increase of body weight (Ryu and Lee, 2021). Therefore, the strong ground reaction after jumping and landing may act not only on the lower limb arch but also on the ankle. The lower limbs of amateur Taekwondo players are the most common injury parts, while the head of professional players is the most common injury part. Epidemiological investigations have found that Taekwondo athletes will have sports injuries such as laceration, contusion, hematoma, cartilage injury, or fracture during the Olympic Games. This is not surprising because Taekwondo athletes usually lack protection for their lower limbs, and the landing after kicking and jumping may transmit large impact and reaction forces (Gartland et al., 2001; Jeong et al., 2019).

Previous studies have found that the distribution pattern of subchondral bone mineral density is considered to reflect stress distribution on the joint surface and subchondral bone under long-term stress loading. Computed tomography osteoabsorptiometry (CTOAM) technology can not only obtain the distribution form of intra-articular mechanical stress but also evaluate the changes of joint mechanical stress after surgery or injury. As a basic clinical research method, this technology is not invasive and has the characteristics of high efficiency and safety for subjects (Müller-Gerbl et al., 1989). At present, there is a lack of image anatomical research on the changes in bone morphology and bone tissue of athletes’ lower limbs under Taekwondo stress loading. Therefore, the purpose of this study is to analyze the distribution pattern of the high-density area of subchondral bone, the volume percentage of bone tissue, and bone remodeling characteristics of the subchondral bone of the ankle in the two groups by using CTOAM. Let us assume that the impact stress, ground reaction force, intra-articular stress, lower limb movement technology, and lower limb muscle and tendon stress caused by Taekwondo lead to special pressure distribution patterns and bone tissue remodeling in the ankle.

## Methods

### Data Collection

The study has been approved by the institutional ethics review committee of the North University of China. Informed consent to participate in the study was obtained from each participant. The height, weight, age, and training history of 10 male Taekwondo athletes (Taekwondo group; Taekwondo National Championship) and 10 male non-Taekwondo athletes (control group) were investigated (Table 1). All Taekwondo athletes have more than 8 years of training history. All subjects volunteered to participate in the experiment and underwent lower extremity CT image scanning (LightSpeed VCT, GE Healthcare, Waukesha, WI, United States; tube voltage: 120 KV, tube current: 250 mA; slice thickness: 0.625 mm). This study has excluded any foot and ankle diseases, discomfort symptoms, and trauma history.

**TABLE 1:** Basic characteristics of subjects.

	Control	Taekwondo	*p* value
Number	10	10	
Dominant foot	Right	Right	
Age (yrs)	20.80 ± 0.92	21.10 ± 0.88	0.406
Height (cm)	178.90 ± 2.63	179.00 ± 2.63	0.933
Weight (kg)	67.30 ± 2.21	67.40 ± 2.27	0.922
Training age (yrs)	0	≥8	

Data are expressed as mean ± SD.

### Computed Tomography Osteoabsorptionmetry

The obtained CT image data were exported in DICOM format and transmitted to Material Mimics 21.0 (Materialise, Leuven, Belgium) for postprocessing. Then, we took the high-density area on the articular surface of the distal tibia and the high-density area on the surface of the talar dome as the region of interest (ROI), and each density area was defined as Hounsfield Unit (HU, which defined as X-ray attenuation; water is 0, and dense bone is 1000). The range of bone mineral density in the articular surface of the distal tibia and that in the subchondral bone of the talus was divided into 11 equal intervals, and each interval was given different colors: black indicated high density and purple indicated low density (Müller-Gerbl et al., 1989; Onodera et al., 2012). Finally, we stacked the bone mask data of each interval to obtain the mapping image of each bone density interval of the whole subchondral bone of the ankle.

### Distribution Patterns of Bone Tissue With Different Densities Within the Subchondral Bone of the Ankle Joint

First, by the regional growth function in Mimics software, the bone masks of each density interval of the distal tibia and subchondral bone of the talus were extracted, and then the extracted bone masks of each density interval were successively reconstructed into the corresponding bone tissue model. Second, the bone tissue models of the distal tibia and subchondral talus were applied with visible function and transparency function (low and medium bone density bone tissue has high transparency; high bone density has medium transparency). Finally, in order to determine the distribution pattern of different bone density bone tissue within the subchondral bone of the ankle joint in the two subjects, we took three tomographic image sections, sagittal, coronal, and axial (medial, middle, and lateral; anterior, middle, and posterior; superior, middle, and inferior), on the articular surface of the distal tibia and the articular surface of the talus in each of the two subjects and observed the distribution of bone tissue of different bone densities within the distal tibia and talar articular surfaces in the computed tomography images.

### Evaluation of the Distribution Pattern of High Bone Density Areas on the Ankle Joint Surface

In order to divide the distribution pattern of subchondral bone high bone density areas on the articular surface of the distal tibia, we divided the reconstructed bone tissue model of the articular surface of the distal tibia and talar dome into nine anatomical regions (Raikin et al., 2007; Elias et al., 2009) to determine the frequency, distribution pattern, and quantity percentage of high bone density areas on the articular surface of the distal tibia and talar dome.

### Quantitative Analysis of the Volume Percentage of the Ankle Subchondral Bone

**FIGURE 1:**
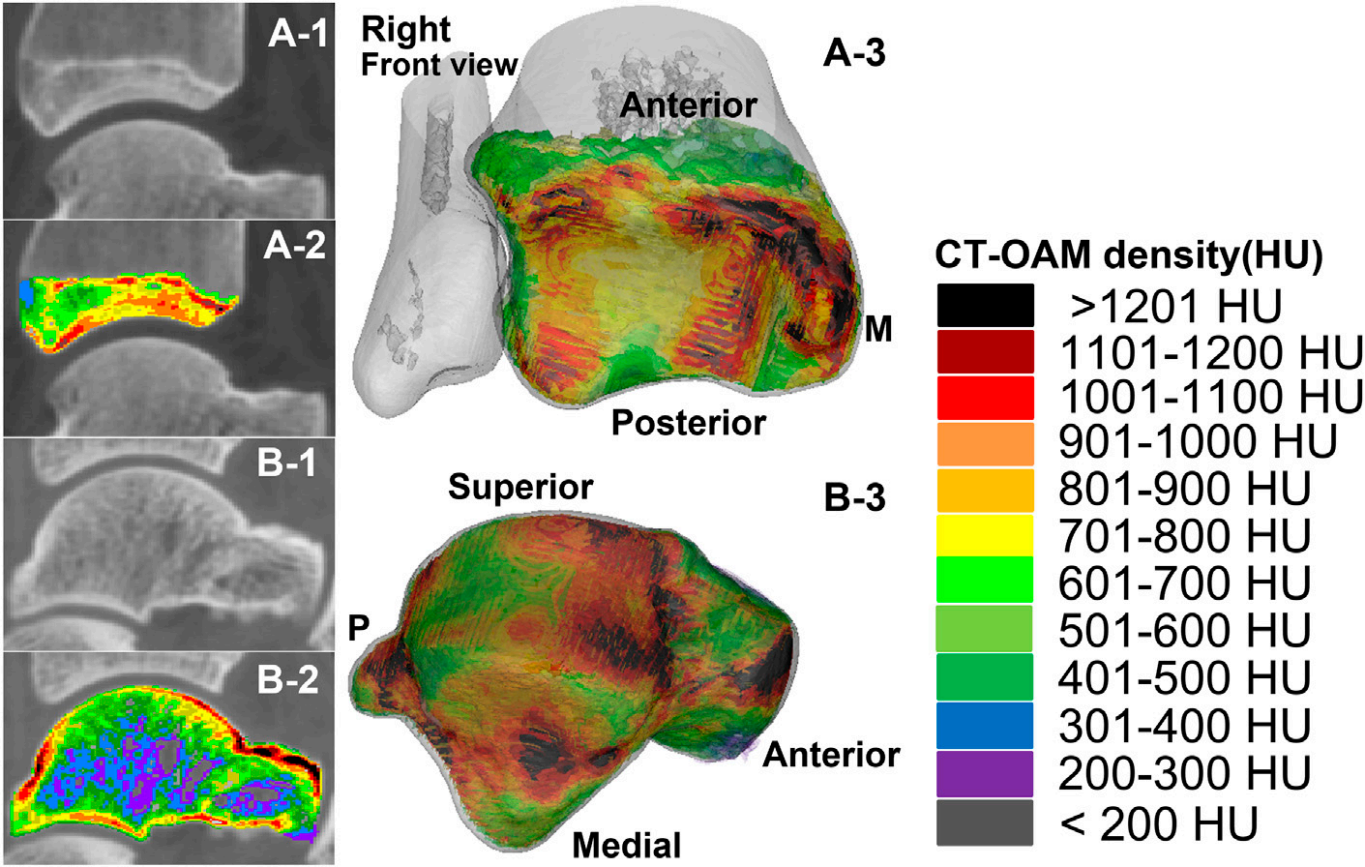
The measurement range and reconstruction model of the subchondral bone of the distal tibia and the talus in the two groups of subjects. A-1 and A-2 were computed tomography (CT) images and CTOAM images of the distal tibia; A-3 is the reconstructed image of each bone density tissue of the distal tibia. B-1 and B-2 were computed tomography (CT) images and CTOAM images of the talus; B-3 is the image of bone density tissue of the talus after reconstruction.

In the images, we took the volume of bone tissue of distal tibia (growth plate to the articular surface) and subchondral bone tissue of talus as the volume of bone tissue of interest and compared and analyzed the volume percentage difference of bone tissue of distal tibia and subchondral bone tissue of the talus between the two groups (bone tissue volume percentage, % BTV). First, we reconstructed the bone mask between the distal tibial growth plate and the articular surface and the bone mask between the subchondral bone of the talus into a three-dimensional model (Figure 1) and derived the bone tissue volume data of each density interval. Second, sum the volume of the distal tibia and subchondral bone tissue of the talus in each bone density interval to obtain the total volume of the distal tibia and subchondral bone tissue of the talus. Finally, the %BTV in the ankle joint surface is obtained through calculation. The formula is as follows:

$$\%BTV=\frac{volume\;of\;bone\;tissue\;with\;a\;certain\;density\;in\;distal\;tibia\;or\;talus}{total\;volume\;of\;subchondral\;bone\;tissue\;of\;distal\;tibia\;or\;talus}.$$

### Statistical Analysis

The measurement results were first tested by Kolmogorov–Smirnov to see whether the data of the two groups conformed to normal distribution. If the data conformed to normal distribution, Student’s *t*-test was used to compare the experimental and control groups. If the distribution did not conform to normal distribution, then the Mann–Whitney *U*-test was used for comparison between groups, the Wilcoxon signed rank test was used for comparison within the experimental group, and statistical results were expressed as mean ± SD, with *p* < 0.05 as statistically significant. IBM SPSS Statistics 25.0 software was used.

## Results

### Comparison of Basic Characteristics Between Groups

The characteristics of the subjects are shown in Table 1. There was no significant difference in age, height, and weight between the two groups of subjects.

### Assessment of the Distribution Pattern of Bone Tissue With Different Densities Under the Cartilage of the Ankle Joint

**FIGURE 2:**
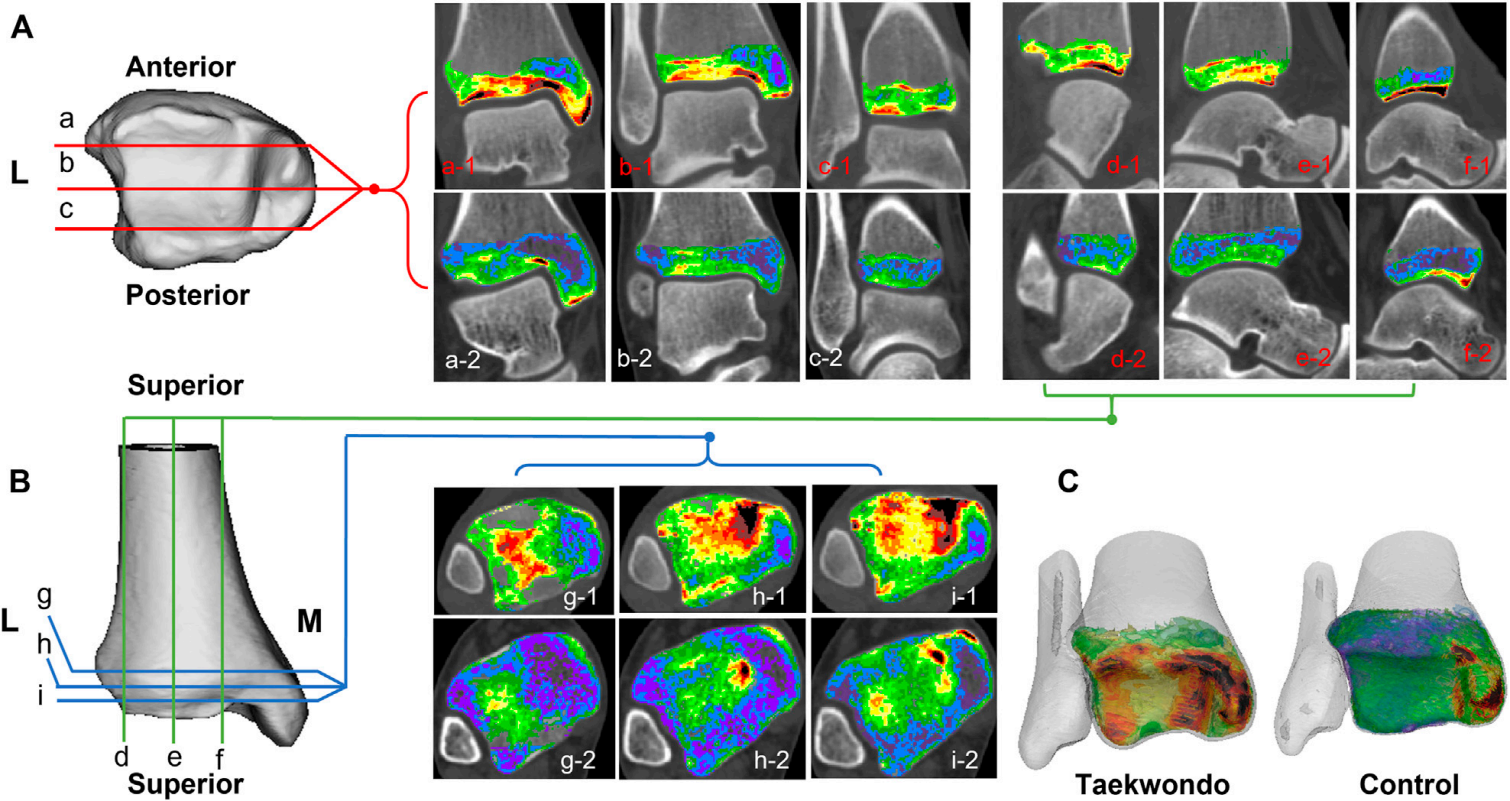
Computed tomographic images of the distribution of different bone density bone tissues in the distal tibia in two groups. **(A)** is a schematic diagram of the coronal tomographic images of the tibia; **(B)** is a schematic diagram of the sagittal and axial tomographic images of the talus; **(C)** is a schematic diagram of the superimposed bone tissue of each bone density within the distal tibia. a1–c1 are the coronal images of the Taekwondo; d1–f1 are the sagittal images of the Taekwondo; g1–i1 are the axial images of the Taekwondo; a2–c2 control coronal images; d2–f2 control sagittal images; g2–i2 control axial images.

**FIGURE 3:**
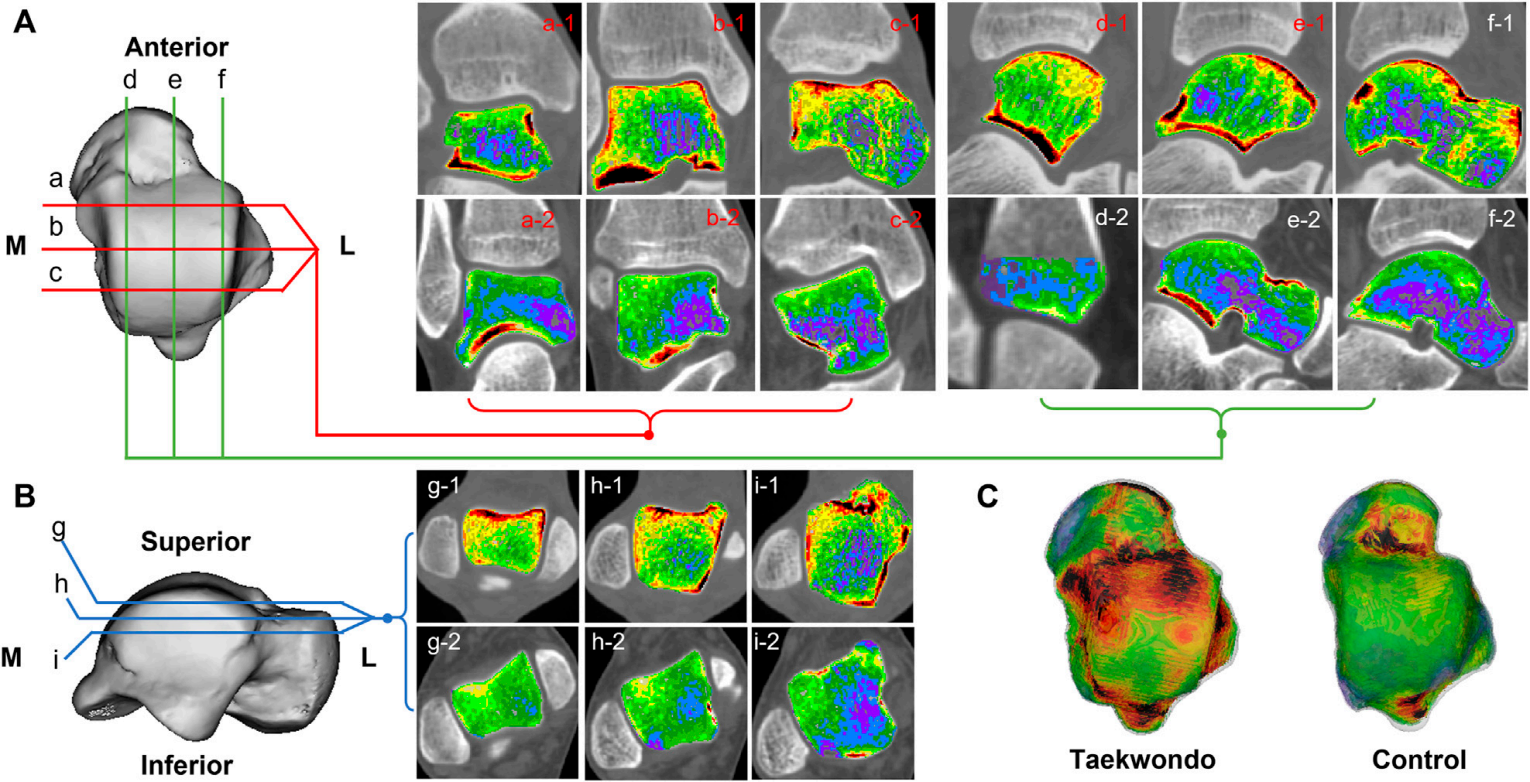
Computed tomographic images of the distribution of bone tissue of different bone densities in the talus of two groups. **(A)** is a schematic diagram of the coronal and sagittal tomographic images of the talus; **(B)** is a schematic diagram of the axial tomographic images of the talus; **(C)** is a schematic diagram of the superimposed bone tissue of each bone density in the talus. a1–c1 are the coronal images of Taekwondo; d1–f1 are the sagittal images of Taekwondo; g1–i1 are the axial images of Taekwondo; a2–c2 control group coronal images; d2–f2 control group sagittal images; g2–i2 control group axial images.

In the sagittal, coronal, and axial images, the bone tissue of each bone density in the distal tibia and talus showed a mixed lamellar–concentric distribution in both groups of subjects, and the bone tissue density was higher on the side close to the articular surface in Figures 2 and 3.

### Comparison of Frequency and Distribution Patterns of the High-Density Zone of the Distal Tibia and Talus Dome Articular Surface in the Three-Dimensional Mode

**FIGURE 4:**
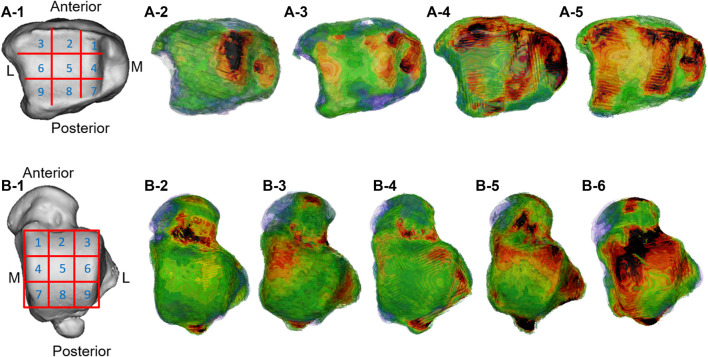
Distribution pattern of the high-density zone of the distal tibial articular surface and talar talus. A-1 and B-1 are the division pattern of the high-density zone of the distal tibial articular surface and talar talus; A-2 was the medial center; A-3 was the medial–lateral center; A-4 was the ring; A-5 was comprehensive; B-2 was blank; B-3 was the medial center; B-4 was lateral center; B-5 was medial–lateral center; B-6 was ring type.

The high-density measurement patterns of the two groups of subjects are shown in Figure 4, and the location and distribution patterns of the high-density areas of the distal tibia and the talar dome surface of the two groups are further analyzed in Table 2.

**TABLE 2:** Distal tibial articular surface and talus dome high-density area classification data.

Position	Distal tibia				Talar dome			
Groups	Control group		Taekwondo		Control group		Taekwondo	
High-density area	Number (20)	Frequency (%)	Number (20)	Frequency (%)	Number (20)	Frequency (%)	Number (20)	Frequency (%)
1	17	85%	20	100%	15	75%	18	90%
2	2	10%	15	75%	11	55%	14	70%
3	4	20%	20	100%	12	60%	18	90%
4	12	60%	19	95%	7	35%	16	80%
5	3	15%	6	30%	2	10%	6	30%
6	5	25%	13	65%	7	35%	18	90%
7	13	65%	20	100%	5	25%	14	70%
8	5	25%	17	85%	1	5%	4	20%
9	4	20%	14	70%	7	45%	15	75%
Blank	0	0	0	0	4	20%	0	0
Medial center	12	60%	0	0	4	20%	0	0
Lateral center	0	0	0	0	2	10%	0	0
Medial–lateral center	7	35%	0	0	10	50%	8	40%
Ring	1	5%	14	70%	0	0	12	60%
Comprehensive	0	0	6	30%	0	0	0	0

In the present study, it was found that there were high-density areas in the 1st, 2nd, 3rd, 4th, 6th, 7th, and 9th regions of the distal tibia of Taekwondo athletes, and the distribution track was consistent with the high-density areas of the talar dome surface (1st, 2nd, 3rd, 4th, 6th, 7th, and 9th regions).

### Comparison of the Volume Percentage of the Bone Density of the Distal Tibia Epiphysis and Talus Subchondral Bone Between the Two Groups

**FIGURE 5:**
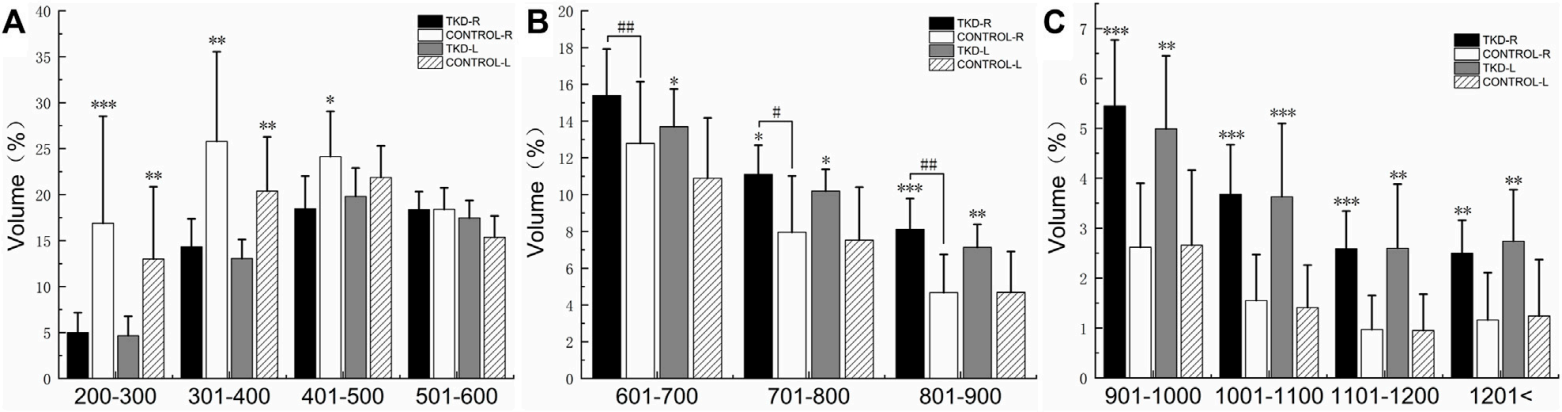
Comparison of the bone volume of each bone density zone in the distal tibia subchondral bone between the groups (%). **(A)** is the bone volume of low bone density; **(B)** is the bone volume of medium bone density; **(C)** is the bone volume of high bone density (***, **, and * indicate statistically significant *p* values of <0.001, <0.01, and <0.05 vs. control group; ^**##**^ and ^**#**^ indicate statistically significant *p* values of <0.01 and <0.05 in Taekwondo in vivo, respectively).

**FIGURE 6:**
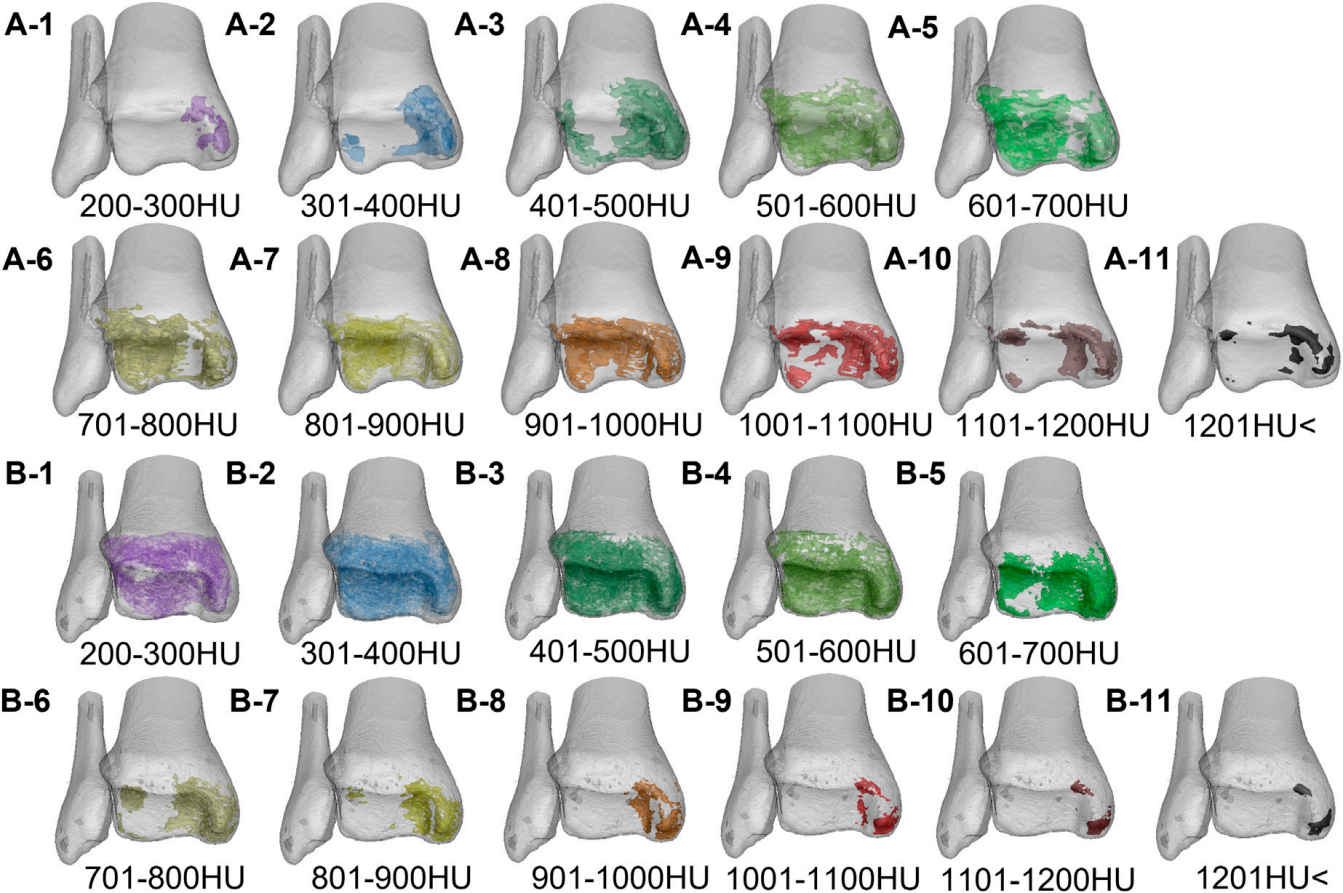
The volume distribution of bone tissue with different bone densities in the distal tibia of the two groups. A1–A11 is the Taekwondo group; B1–B11 is the control group.

%BTV of the moderate and high bone mineral density of subchondral bone of distal tibia in the Taekwondo group was higher than that in the control group (*p* > 0.05). The right distal tibia of the Taekwondo group had a higher %BTV of moderate bone mineral density in the subchondral bone than the left distal tibia of the Taekwondo group (*p* > 0.05). %BTV of low bone density bone tissue of distal tibial subchondral bone in the control group was higher than that in the Taekwondo group (*p* > 0.05) in Table 3 and Figures 5 and 6.

**TABLE 3:** Comparison of the bone volume of each bone density zone in the distal tibia subchondral bone between the groups (%).

Density classifications	Unit:HU	Right		*p* value	Left		*p* value
		Control	Taekwondo		Control	Taekwondo	
Low bone density	200–300	16.90 ± 11.63	4.99 ± 2.17	**0.001**	13.01 ± 7.83	4.66 ± 2.10	**0.008**
	301–400	25.79 ± 9.75	14.33 ± 3.04	**0.005**	20.38 ± 5.89	13.06 ± 2.08	**0.003**
	401–500	24.13 ± 4.93	18.47 ± 3.55	**0.011**	21.87 ± 3.44	19.81 ± 3.07	0.174
	501–600	18.39 ± 2.36	18.36 ± 1.96	0.974	15.37 ± 2.32	17.48 ± 1.87	0.105
Medium bone density	601–700	12.79 ± 3.35	15.39 ± 2.53^**##**^	0.066	10.89 ± 3.28	13.70 ± 2.04	**0.033**
	701–800	7.96 ± 3.06	11.10 ± 1.59^**#**^	**0.012**	7.53 ± 2.87	10.19 ± 1.19	**0.019**
	801–900	4.67 ± 2.08	8.12 ± 1.66^**##**^	**0.001**	4.69 ± 2.22	7.14 ± 1.24	**0.009**
High bone density	901–1000	2.62 ± 1.28	5.45 ± 1.32	**0.001**	2.66 ± 1.50	4.99 ± 1.46	**0.002**
	1,001–1,100	1.55 ± 0.92	3.68 ± 0.99	**0.001**	1.41 ± 0.85	3.63 ± 1.47	**0.001**
	1,101–1,200	0.97 ± 0.68	2.59 ± 0.75	**0.001**	0.95 ± 0.73	2.60 ± 1.28	**0.002**
	1,201–maximum	1.16 ± 0.95	2.50 ± 0.66	**0.004**	1.24 ± 1.13	2.74 ± 1.03	**0.006**

The statistical results are expressed as mean ± SD. ^##^ and ^#^ indicate statistically significant *p* values of <0.01 and <0.05 in Taekwondo vivo, respectively. %BTV: percentage change in bone tissue volume for each bone density within the distal tibial subchondral bone.

To indicate that the data comparisons are different, we put boldface on the P values that are significantly different.

**FIGURE 7:**
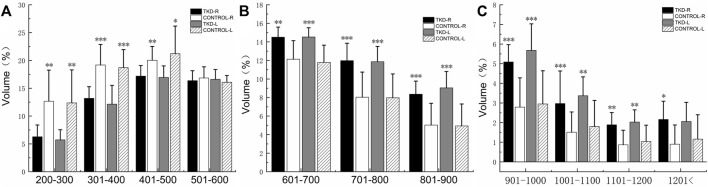
Comparison of the bone volume of each bone density zone in the talus subchondral bone between the groups (%). **(A)** is the bone volume of low bone density; **(B)** is the bone volume of medium bone density; **(C)** is the bone volume of high bone density (***, **, and * indicate statistically significant *p* values of <0.001, <0.01, and <0.05 in the control group).

**FIGURE 8:**
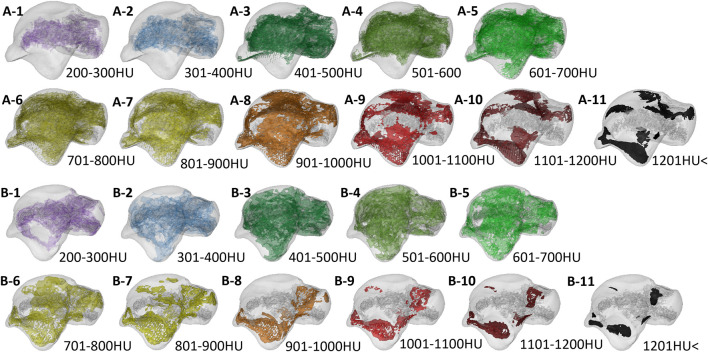
The volume distribution of bone tissue with different bone densities in the talus subchondral bone of the two groups. A1–A11 is the Taekwondo group; B1–B11 is the control group.

%BTV with the moderate and high bone density of subchondral bone of talus in the Taekwondo group was higher than that in the control group (*p* > 0.05). %BTV with the low bone density of subchondral bone of talus in the control group was higher than that in the Taekwondo group (*p* > 0.05) in Table 4 and Figures 7 and 8.

**TABLE 4:** Comparison of the bone volume of each bone density zone in the talus subchondral bone between the groups (%).

Density classifications	Unit: HU	Right		*p* value	Left		*p* value
		Control	Taekwondo		Control	Taekwondo	
Low bone density	200–300	12.67 ± 5.59	6.28 ± 2.12	**0.006**	12.37 ± 5.92	5.73 ± 1.81	**0.006**
	301–400	19.18 ± 3.69	13.19 ± 2.09	**0.001**	18.70 ± 3.23	12.14 ± 3.38	**0.001**
	401–500	20.03 ± 2.48	17.18 ± 1.89	**0.010**	21.21 ± 4.96	16.95 ± 2.06	**0.022**
	501–600	16.84 ± 2.01	16.37 ± 1.78	0.584	16.08 ± 1.20	16.61 ± 1.76	0.448
Medium bone density	601–700	12.13 ± 2.00	14.51 ± 1.08	**0.005**	11.79 ± 1.85	14.53 ± 0.99	**0.001**
	701–800	8.04 ± 2.70	11.98 ± 1.87	**0.001**	7.97 ± 2.57	11.86 ± 1.65	**0.001**
	801–900	5.03 ± 2.35	8.36 ± 1.41	**0.001**	4.94 ± 2.36	9.04 ± 1.76	**0.001**
High bone density	901–1000	2.79 ± 1.49	5.09 ± 0.88	**0.001**	2.95 ± 1.69	5.68 ± 1.35	**0.001**
	1,001–1,100	1.51 ± 1.03	2.97 ± 1.66	**0.001**	1.80 ± 1.33	3.37 ± 0.96	**0.007**
	1,101–1,200	0.87 ± 0.74	1.89 ± 0.62	**0.003**	1.04 ± 0.83	2.03 ± 0.62	**0.008**
	1,201–maximum	0.90 ± 0.98	2.16 ± 0.93	**0.019**	1.16 ± 1.24	2.06 ± 0.97	0.052

The statistical results are expressed as mean ± SD. %BTV: percentage change in bone tissue volume for each bone density within the talus subchondral bone.

To indicate that the data comparisons are different, we put boldface on the P values that are significantly different.

## Discussion

### The Stress Distribution of Ankle Joint Surface Between Taekwondo and Control Subjects Was Different

In the plantar flexion–internal rotation of the ankle, the posteromedial region of the talus contacts the distal tibia. When the ankle is dorsiflexion, the talus slides posteriorly toward the tibia. In ankle plantar flexion, the talus slides forward to the tibia (Stormont et al., 1985; Hayes et al., 2006; Kobayashi et al., 2015). Therefore, as the ankle moves from dorsiflexion to plantar flexion, the contact trajectory between the talus and the tibia is posteriorly anterior to the tibia (Driscoll et al., 1994; Omori et al., 2004). In the aforementioned experiments, cadaver specimens were used to measure the difference in stress distribution and joint motion pattern of the distal tibia. *In vivo*, bone joints are also filled with bone joint fluid to lubricate them, helping them to complete rolling, friction, impact, sliding, and other single and composite actions. In the present study, it was found that there were high-density areas in the 1st, 2nd, 3rd, 4th, 6th, 7th, and 9th regions of the distal tibia of Taekwondo athletes, and the distribution track was consistent with the high-density areas of the talar dome surface (1st, 2nd, 3rd, 4th, 6th, 7th, and 9th regions). In addition, we also noticed that some Taekwondo athletes had higher bone density distribution in the second region of the talus and talus articular surface. Therefore, we believe that Taekwondo athletes’ take off movement in a short period of time, and gravity reaction force after landing, lower limb mobility technology, and long-term impact stress loading lead to the high-density distribution of the distal tibial articular surface and talar dome area. Finally, we also observed several unreported distribution types of high-density areas (Leumann et al., 2015; Deml et al., 2017; Shiota et al., 2020), such as blank area and annular high-density area on the talar dome of subjects in the control group and Taekwondo group, and annular and comprehensive high-density area on the distal tibia. We suggested that the control subjects may not have a long-term or high-intensity exercise habit and therefore do not develop a large area of high density in the medial to lateral area of the talar dome surface. Differences in the distribution of medial–lateral high-density in the distal tibia and talus cartilage may also be related to the subjects’ gait cycle, skeletal morphology, joint stress pattern, genetics, and shoe type. In addition, according to the frequency of high-density areas on the articular surface of the distal tibia and talar dome surface, the 1st, 4th, and 7th regions of the normal stress part of the distal tibia and the 1st, 4th, and 9th regions of the talar dome surface were selected as the reference sites in the control group. The stress position of the distal tibial articular surface and talar dome surface of Taekwondo athletes is based on the normal stress area. After long-term stress loading of the joint, part of the middle region and part of the lateral region (low-stress area) high-density area increases, that is, the ankle joint surface of Taekwondo athletes shows a whole stress state.

Generally speaking, from the perspective of functional anatomy, the joint surfaces of the ankle move with each other in the form of extrusion, collision, and friction, which can lead to obvious contact stress distribution on the joint surfaces of the two. This change is very consistent with the report that the contact pressure distribution of the ankle joint moves from the middle to the anterior region of the talar dome surface from the first peak to the second peak of the standing stage. During the standing period of ankle gait, the contact stress on the talar dome surface mainly concentrates on the middle region of the talar dome surface. The maximum load was detected in the anterolateral region of the talar dome during the thrust phase of the ankle gait. During the transition from plantar flexion to dorsiflexion, the lateral load of the talus increases and the medial load decreases (Michelson et al., 2001; Suckel et al., 2010; Park et al., 2019). In addition, the range of plantar flexion of the ankle joint is 40–50°, while the range of dorsiflexion is about 20°. The 20° dorsiflexion range left by Taekwondo athletes after jumping and landing may lead to the high-density regional distribution phenomenon in the 1st, 2nd, 3rd, 4th, 6th, 7th, and 9th regions of the talus. In the field of sports medicine research, some scholars think that there was a higher risk of cartilage damage lesions in the medial region 4th and lateral region 6th of the talar dome surface and region 4th on the medial distal side of the tibia, and main factors of cartilage damage may be due to form a violent collision between two bone, articular cartilage, as a result of increased ankle internal stress loading damage (Raikin et al., 2007; Elias et al., 2009). Generally speaking, the ankle can cushion the impact of landing. However, some studies have found that depending on the height of the jumping landing, the ankles of subjects need to bear 6–10 times the weight of ground reaction force when their legs land, resulting in high-intensity vibration of the joints (Zhang et al., 2000; Ryu and Lee, 2021). The number of ground reaction forces varies with the type of ballet technique (McPherson et al., 2019). And, the ground reaction force explains the force exerted by the ankle joint on the ground and the feedback of that force. Most lower limb injuries in athletes occur after landing on one leg, and the ankle joint is unable to withstand the impact stress of landing (Olsen et al., 2004; Ryu and Lee, 2021). This suggests that Taekwondo athletes may be potential patients with an ankle cartilage injury. Therefore, it is recommended that Taekwondo athletes receive regular medical imaging during training to reduce the potential risk of cartilage damage.

Compared with the experimental results of Shiota et al. (2020), the distribution pattern of the high-density area of the tibial ankle joint of Taekwondo athletes is similar to that of soccer players. Although soccer and Taekwondo belong to lower limb sports, soccer players have a shorter time dribbling the ball and running distance, while running without the ball more time. Soccer players, however, wear professional sneakers to play on grass, which makes it possible to cushion the ankle to some extent. In addition, when Taekwondo athletes wear electronic protective gear on their lower limbs, they only get the score of hitting, but it does not provide much protection to the feet of Taekwondo athletes. As a result, large areas of high bone density are distributed in the ankle joint of Taekwondo athletes.

### The Mechanical Stress of Taekwondo Promotes the Difference in Ankle Bone Remodeling Distribution

In the sagittal, coronal, and axial images, the bone tissue of each bone density in the distal tibia and talus showed a mixed lamellar–concentric distribution in both groups of subjects, and the bone tissue density was higher on the side close to the articular surface. Compared with the control group, the moderate and high bone volume of ankle joint subchondral bone in the Taekwondo group was significantly increased. It indicates that long-term stress loading results in a high degree of remodeling of the subchondral bone of the ankle joint of athletes, and the remodeling position of the bone is located in the stress position of the joint. According to the age and training history of the subjects, the participants of the high-level Taekwondo athletes have more than 8 years of training history on average. Moving the training history forward to the beginning of the training period, the mechanical stress stimulation resulting from Taekwondo training or competition at the same age may accelerate the pubertal bone development and remodeling process in athletes. After Taekwondo athletes take off and hit the target, their bodies must receive 6 times of their own ground reaction force after landing. If they land on one foot and gain weight, the risk of lower limb injury will be further increased (Ryu and Lee, 2021). The athlete keeps the jump-landing posture during training and competition, which causes the ankle, knee, and hip joints to continuously suffer from the body and ground gravity and ground reaction force, resulting in the collision between the talus and the distal tibia, resulting in the ankle surface bone remodeling phenomenon. According to Wolfe’s law (Wolff, 2010) and research findings, long-term high-intensity exercise stress may lead to adaptive changes in bone (Lara et al., 2016). Impact exercise intervention in adult women found ossification in the distal tibia, while resistance training had a greater impact on the strength of the femoral shaft and proximal femur (Lambert et al., 2020).

Early studies have found that subchondral ossification of the distal end of long bones over time may indicate a functional adaptation of the distal tibial articular surface to joint load, but may also lead to articular cartilage damage and osteoarthritis (Muir et al., 2006). However, recent studies have found that the bone mineral density of the spine and lower limb (vertebral body, femoral neck, and femoral trochanter) of retired soccer players is significantly higher than that of the control group, and the prevalence of knee osteoarthritis is lower than that of nonathletes, suggesting that low bone mineral density and advanced age are the main influencing factors of knee osteoarthritis (Lv et al., 2018). In Taekwondo athletes after early training, the volume remodeling level of the distal tibia and subtalus bone tissue is constantly improved to resist stress stimulation. Some studies have found that, with the change in biomechanical environment, the modeling and remodeling of subchondral bone is faster, while the conversion rate of articular cartilage is relatively low (Intema et al., 2010; Goldring, 2012; Zhen and Cao, 2014). In general, subchondral bone has greater stiffness and strength than articular cartilage and can absorb much of the stress transferred from the articular surface and provide biomechanical support to cartilage (Zhen and Cao, 2014). Therefore, we can know that reducing the stress carrying capacity of subchondral bone (bone density) can lead to changes in the stress distribution of articular cartilage, thus increasing the probability of articular cartilage injury. Studies have found that patients with osteoporosis and osteoarthritis of subchondral bone remodeling can lead to serious abnormal cartilage damage, and abnormal subchondral bone remodeling damaged the normal biomechanical environment, exacerbating the cartilage injury risk; this shows that the stress load transfer mechanism in the subchondral bone and articular cartilage plays a key role in the functional unit (Chu et al., 2019). Subchondral bone remodeling with a good stress transfer effect may reduce the high-intensity stress carried by articular cartilage. However, it is puzzling that Taekwondo athletes did not show any ankle injury or ankle pain symptoms before the CT scan. This may be related to the arch of the foot and the muscles and ligaments around the ankle joint of Taekwondo athletes, which buffer the stress and provide support and nutrition for the ankle joint (Guimarães et al., 2018; Rice et al., 2019; Sutter et al., 2019), thus reducing the probability of joint injury to a certain extent.

### Mechanical Stress in Taekwondo Promotes the Distribution of High-Density Bone in Other Parts of Ankle Joint

The current study demonstrates that, outside the zone of interest at the distal tibia and talar dome, we noted areas of high density at the tibial medial malleolus tip and the head of the talus, the lateral tuberosity, and the outer ankle surface. We thought the Taekwondo athletes after landing, ankle joint instability there may be a moment in (including ankle inversion and eversion action in the coronal plane), ankle plantar flexion and backstretch displacement, landing back within the asymmetric stress and arch deformation after extrusion (e.g., from the boat joints), the surrounding ligament pull on the ankle, and leading to the aforementioned areas appear large bone remodeling phenomenon. Compressive strain, tensile strain, hydrostatic pressure, shear strain, and hydrodynamics are considered to be important mechanical loading cues that regulate bone regeneration and remodeling (Goodship and Kenwright, 1985; Carter et al., 1988; Leucht et al., 2007; Hara et al., 2018). Earlier studies found that when subjects landed on one side, they increased the range of motion of the ankle joint, the impact absorption of the surrounding muscle tissue, the risk of injury, and the total energy absorption after landing by 11%, suggesting that there was a significant amount of passive energy transferred from both sides during a one-sided landing (Weinhandl et al., 2010). Athletes with chronic ankle instability had a significant ankle valgus angle at initial landing, suggesting a preference for additional ankle valgus angles during landing to avoid ankle sprain due to ankle rollover (Lin et al., 2019). After Taekwondo athletes take off and land, the instantaneous instability of the ankle joint in the coronal plane (including ankle varus and valgus) may cause the tension of ligaments attached to the inside and outside of the ankle joint to be pulled alternately. Bone remodeling was caused by the impact of the fibula lateral malleolus tip and talar dome, as well as the medial malleolus tip and talus lateral malleolus surface of the distal tibia. The high-density area and bone remodeling of the talus and lateral tuberosity may be caused by the intercompression of the talus navicular bone joints in the arch of the foot, the mutual pulling of the talus and navicular bone by the talus dorsal ligament, the intercompression of the calcaneal posterior articular surface and the posterior talus joint, and the tension of the talus, and the calcaneus posterior ligament. Early studies using MRI resonance found that during the training period of military recruits, a large number of bone stress injuries occurred in the talus of the foot of lower limbs, and all of them were concentrated in the upper region of the talus (Sormaala et al., 2006). However, it has been found that an increase in hindfoot valgus and a decrease in forefoot valgus may exacerbate the shear stress in the middle third of the navicular bone. If the forefoot valgus angle is reduced, the pressure on the talus increases. The medial rotation of the talus causes the talus to exert force on the lateral side of the navicular bone. However, it has been found that an increase in hindfoot valgus and a decrease in forefoot valgus may exacerbate the shear stress in the middle third of the navicular bone as posterior valgus is increased (Arndt et al., 2007; Becker et al., 2018). The findings of the present study are consistent with those reported by Mueller et al. (2014). In the three-dimensional model of the talus and the two-dimensional images of the coronal surface of the navicular bone, high-density zones were observed in the posterior articular surface of the talus and in the talonavicular surface. Based on our current results, we conclude that the interaction between the talus and navicular bone can be used to transfer and release the intra-articular stress generated by the human arch of the talus. However, it has been found that an increase in hindfoot valgus and a decrease in forefoot valgus may exacerbate the shear stress in the middle third of the navicular bone. The talus–navicular joint pulls the talonavicular ligament during movement, resulting in a high-density distribution of the head of the talus. The high-density distribution of the external ankle surface of the talus is mainly caused by the interaction between the internal fibula surface and the external talus ankle surface, the friction of the calcaneal ligament, and the traction of the talocalcaneal lateral ligament. In addition, the posterior articular surface of the calcaneus and the talus forms the subtalar joint, and the plantar-flexion motion of the calcaneus pulled by the Achilles tendon results in the interaction between the posterior articular surface of the calcaneus and the talus, resulting in a high-density distribution of the posterior articular surface of the talus. The mechanical relationship between the talar head and the navicular bone remains unclear and needs to be further investigated by bone and joint biomechanics and finite element analysis of cadaveric specimens.

## Limitation

For the current studies, this study has several limitations. 1) The corresponding biomechanical experiment was not carried out in this test, and the stress parameters generated by Taekwondo athletes’ striking and footwork movement were not obtained. 2) As far as the present study is concerned, the experiment fails to determine which direct or indirect factors will lead to the high-density stress distribution of the distal tibial bone of the ankle joint of Taekwondo athletes.

## Conclusion

The impact stress, ground reaction force, intra-articular stress, lower limb movement technology, lower limb muscle, and tendon stress caused by Taekwondo lead to special pressure distribution patterns and bone tissue remodeling in the ankle. The distal tibia and talus bone transformation rule for low density in bone tissue to medium density bone tissue to high-density and medium-density bone tissue. The stress points on the articular surface of the ankle joint are the 1st, 4th, and 7th regions of the distal tibia, and the 1st, 3rd, 4th, and 7th regions of the talar dome surface. The ankle joint of Taekwondo athletes is comprehensively stressed based on the normal stress point, to improve the area of high density near the area of low stress. Because Taekwondo athletes have a large area of high-density distribution and high bone remodeling on the distal tibia and talus joint surface, we suggest that coaches and athletes should receive CT images and MRI examinations regularly to observe the wear of athletes’ articular cartilage.

## Data Availability

The raw data supporting the conclusions of this article will be made available by the authors, without undue reservation.
